# Identification of pathogenicity‐related genes in *Fusarium oxysporum* f. sp. *cepae*

**DOI:** 10.1111/mpp.12346

**Published:** 2016-02-23

**Authors:** Andrew Taylor, Viktória Vágány, Alison C. Jackson, Richard J. Harrison, Alessandro Rainoni, John P. Clarkson

**Affiliations:** ^1^ Warwick Crop Centre, School of Life Sciences University of Warwick Wellesbourne Warwick CV35 9EF UK; ^2^ East Malling Research New Road East Malling Kent ME19 6BJ UK; ^3^ Present address: Enza Zaden Via San Leonardo, 13 Tarquinia (Viterbo) c.a.p. 01016 Italy

**Keywords:** effector genes, *Fusarium* basal rot, *Fusarium oxysporum* f. sp. *cepae*, onion, pathogenicity, *Secreted In Xylem (SIX)*

## Abstract

Pathogenic isolates of *Fusarium oxysporum*, distinguished as *formae speciales* (f. spp.) on the basis of their host specificity, cause crown rots, root rots and vascular wilts on many important crops worldwide. *Fusarium oxysporum* f. sp. *cepae* (FOC) is particularly problematic to onion growers worldwide and is increasing in prevalence in the UK. We characterized 31 *F. oxysporum* isolates collected from UK onions using pathogenicity tests, sequencing of housekeeping genes and identification of effectors. In onion seedling and bulb tests, 21 isolates were pathogenic and 10 were non‐pathogenic. The molecular characterization of these isolates, and 21 additional isolates comprising other f. spp. and different *Fusarium* species, was carried out by sequencing three housekeeping genes. A concatenated tree separated the *F. oxysporum* isolates into six clades, but did not distinguish between pathogenic and non‐pathogenic isolates. Ten putative effectors were identified within FOC, including seven *Secreted In Xylem* (*SIX*) genes first reported in *F. oxysporum* f. sp. *lycopersici*. Two highly homologous proteins with signal peptides and RxLR motifs (*CRX1*/*CRX2*) and a gene with no previously characterized domains (*C5*) were also identified. The presence/absence of nine of these genes was strongly related to pathogenicity against onion and all were shown to be expressed *in planta*. Different *SIX* gene complements were identified in other f. spp., but none were identified in three other *Fusarium* species from onion. Although the FOC *SIX* genes had a high level of homology with other f. spp., there were clear differences in sequences which were unique to FOC, whereas *CRX1* and *C5* genes appear to be largely FOC specific.

## Introduction

*Fusarium oxysporum* is a major pathogen of many important crops worldwide, causing crown and root rots as well as vascular wilts (Leslie and Summerell, 2006). The soil‐borne fungus affects a very wide range of crop hosts, including onion, leek, lettuce, tomato, brassicas, asparagus, cucurbits, peppers, coriander, spinach, basil, beans, peas, strawberry, watermelon and banana, and also important non‐food crops, such as carnation and narcissus (Leslie and Summerell, 2006; Michielse and Rep, 2009). Its wide host range, as well as its economic and scientific impact, means that *F. oxysporum* was recently identified as the fifth most important plant‐pathogenic fungus (Dean *et al*., 2012). *Fusarium oxysporum* is a species complex and includes both non‐pathogenic and pathogenic isolates. Non‐pathogenic *F. oxysporum* isolates commonly occur in the soil as saprophytes, while some have been identified as biocontrol agents and endophytes (Alabouvette *et al*., 2009). Pathogenic *F. oxysporum* isolates are distinguished as *formae speciales* (f. spp.) on the basis of their host specificity (Leslie and Summerell, 2006), and more than 120 have been identified (Michielse and Rep, 2009). Recent advances in the understanding of the pathogenicity in *F. oxysporum* have been made following publication of the genome of *F. oxysporum* f. sp. *lycopersici* (FOL), which infects tomato (Ma *et al*., 2010). This led to the discovery of lineage‐specific mobile pathogenicity chromosomes which contain pathogenicity‐related genes. These include *Secreted In Xylem* (*SIX*) genes, the products of which are small effector proteins secreted by FOL during the colonization of tomato plants (Ma *et al*., 2010). So far, 14 *SIX* genes have been identified in FOL, which show no homology with each other or with any other sequenced gene (Houterman *et al*., 2007; Schmidt *et al*., 2013), except for *SIX6* which has homologues in *Colletotrichum* (Gawehns *et al*., 2014). *SIX1* (also known as *Avr3*), *SIX3* (*Avr2*), *SIX4* (*Avr1*) and *SIX5* are recognized by resistance genes which have been introgressed into tomato (Houterman *et al*., 2008, 2009; Ma *et al*., 2015; Rep *et al*., 2004; Takken and Rep, 2010), whereas gene knock‐outs have demonstrated that *SIX1*, *SIX3*, *SIX5* and *SIX6* contribute directly to virulence (Gawehns *et al*., 2014; Houterman *et al*., 2009; Ma *et al*., 2015; Rep, 2005; Takken and Rep, 2010). *SIX* genes have also been found in other f. spp. of *F. oxysporum* (Fraser‐Smith *et al*., 2014; Sasaki *et al*., 2015b), notably *SIX1*, *SIX4*, *SIX8* and *SIX9* in an *F. oxysporum* isolate infecting *Arabidopsis* and *Brassica* (Thatcher *et al*., 2012), *SIX6* in *F. oxysporum* f. sp. *vasinfectum* (Chakrabarti *et al*., 2011), *SIX1* and *SIX6* in f. sp. *betae* (Covey *et al*., 2014), *SIX1*, *SIX7* and *SIX10* in f. spp. *canariensis* and *lini* (Laurence *et al*., 2015), *SIX1, SIX7* and *SIX8* in f. sp. *cubense* (Meldrum *et al*., 2012) and *SIX3*, *SIX5* and *SIX7* in f. sp. *cepae* (Sasaki *et al*., 2015b). In addition, *SIX4* has been shown to play a role in the virulence of *F. oxysporum* f. sp. *conglutinans*, the cause of cabbage yellows (Kashiwa *et al*., 2013).

The genetically heterogeneous nature and lack of reliable morphological characters in the *F. oxysporum* complex have meant that distinguishing between pathogenic and non‐pathogenic isolates and between different f. spp. has been challenging, and has previously relied on pathogenicity tests on different host plants. Molecular methods based on standard approaches, such as DNA fingerprinting and multilocus genotyping using housekeeping genes, have failed to reliably distinguish between different f. spp. (O'Donnell *et al*., 1998), but the presence/absence of certain *SIX* genes or sequence differences in these genes may form the basis for more reliable detection and identification. *SIX* genes have been used to identify and distinguish races in FOL, where race 2 and 3 isolates, which lack the *SIX4* gene found in race 1, are identified on the basis of variation in *SIX3* sequence (Lievens *et al*., 2009). Sequence differences in *SIX8* have also been used to identify and distinguish races of *F. oxysporum* f. sp. *cubense*, including tropical race 4 (Fraser‐Smith *et al*., 2014).

Bulb onion (*Allium cepa* L.) is an important crop globally with a total production of 83 million tonnes (FAOSTAT, 2012). Production is often affected by *Fusarium* basal rot (FBR) caused by *F. oxysporum* f. sp. *cepae* (FOC), which is increasing in prevalence, particularly in the UK (Taylor *et al*., 2013). FOC infects the roots and basal plates of onions, causing symptoms at all stages of plant development, ranging from damping off and delayed seedling emergence to bulb rot at pre‐ and post‐harvest stages (Entwistle, 1990). Infection is favoured by warm temperatures (28–32 °C optimum) and disease incidence is predicted to increase as a result of climate change (Abawi and Lorbeer, 1972; Cramer, 2000; Kehr *et al*., 1962). FOC also produces chlamydospores which can survive for many years in the soil, making disease management very challenging (Brayford, 1996; Cramer, 2000). Other *Fusarium* species have also been associated with root rots of onions or other alliums, including *F. proliferatum*, *F. redolens* and *F. avenaceum*, but are generally less common than FOC (Bayraktar and Dolar, 2011; Du Toit *et al*., 2003; Galván *et al*., 2008; Ghanbarzadeh *et al*., 2013; Shinmura, 2002; Stankovic *et al*., 2007; Yamazaki *et al*., 2013), particularly in the UK (Vágány, 2012).

Few studies have attempted the molecular characterization of FOC isolates specifically, but phylogenetic analyses based on *translation elongation factor 1α* (*EF‐1α*) sequencing and amplified fragment length polymorphism (AFLP) markers have suggested that *F. oxysporum* can be divided into three distinct clades, with FOC isolates appearing in two of these (Galván *et al*., 2008; O'Donnell *et al*., 1998; Sasaki *et al*., 2015b; Taylor *et al*., 2013). A more recent study using the intergenic spacer region (IGS) identified eight clades among FOC isolates from bulb onion and *Allium fistulosum* (Sasaki *et al*., 2015b). However, few molecular studies with FOC, or indeed other f. spp., have associated pathogenicity tests which has confused identification, although a partial association was observed between pathogenicity on Welsh onion and IGS sequence (Dissanayake *et al*., 2009a; Sasaki *et al*., 2015b). Studies have also shown that there is genetic diversity amongst FOC isolates based on AFLP markers, inter‐simple sequence repeat (ISSR) markers, random amplified polymorphic DNA (RAPD) markers, rRNA, *EF‐1α* or IGS sequencing (Bayraktar and Dolar, 2011; Bayraktar *et al*., 2010; Dissanayake *et al*., 2009ba, b; Galván *et al*., 2008; Sasaki *et al.*, 2015b; Southwood *et al*., 2012a, 2012b; Vágány, 2012). More recently, homologues of *SIX3, SIX5* and *SIX7* have been identified in FOC and their presence has been associated with pathogenicity on Welsh onion seedlings (Sasaki *et al*., 2015b). To date, this is the only record of any putative effector genes in FOC.

The aim of this study was to characterize *F. oxysporum* isolates from onion through the sequencing of housekeeping and pathogenicity‐related genes with a particular emphasis on *SIX* genes. The presence/absence of pathogenicity genes was then compared with the ability of the isolates to cause disease in both onion seedlings and bulbs. Isolates of *F. oxysporum* f. spp. *pisi* (pea), *dianthi* (carnation), *narcissi* (daffodil), *cubense* (banana), *lycopersici* (tomato) and other *Fusarium* species isolated from onion/leek (*F*. *avenaceum*, *F*. *proliferatum* and *F*. *redolens*) were also included in the molecular characterization for comparison.

## Results

### Pathogenicity testing

**Figure 1:**
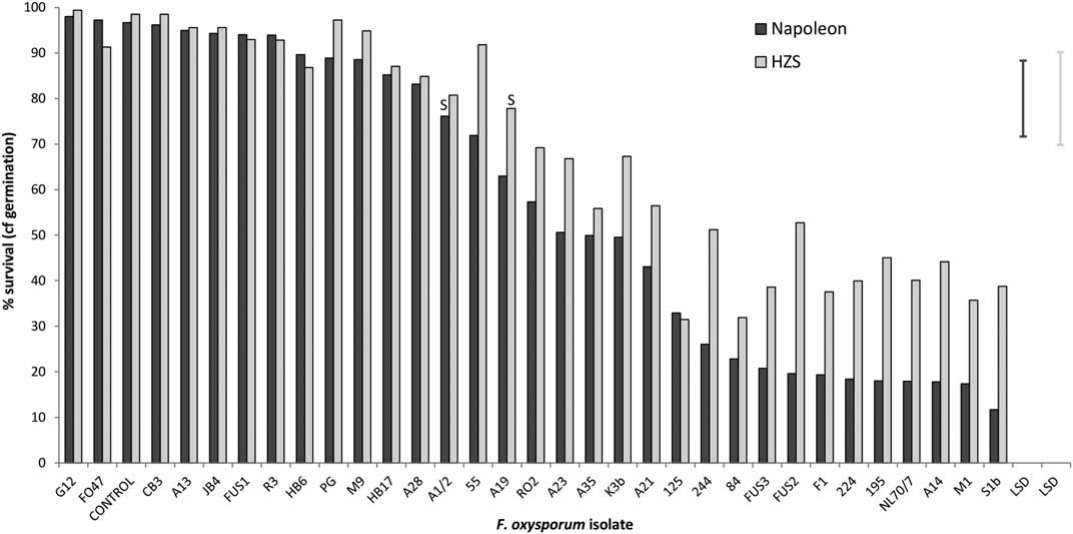
Pathogenicity of 32 *Fusarium oxysporum* isolates on onion seedlings (cv. Napoleon and Hazera Seeds standard susceptible line, HZS). Data shown are the percentage survival values relative to germination after 42 days in a glasshouse. Error bars represent the least significant difference (LSD) (5%) level for each onion cultivar. An ‘S’ indicates the value below which there is a significant difference from control plants.

In the onion seedling tests, significant differences were observed in the pathogenicity of the 32 *F. oxysporum* isolates (Table 1) for the two experiments using Napoleon and HZS onion cultivars (Fig. 1, *P* < 0.001). The pathogenicity of each *F. oxysporum* isolate was highly correlated between the two cultivars (*r* = 0.97, *P* < 0.001). Across both onion cultivars, 18 of the 32 isolates resulted in a significant reduction in seedling survival compared with the uninoculated control and were classed as pathogenic, whereas the remaining 14 isolates (including Fo47) were non‐pathogenic and had little or no effect (Fig. 1). Two isolates (A1_2 and 55) caused significant seedling mortality on cv. Napoleon, but not on HZS. Over all the isolates, Napoleon was more susceptible than HZS to *F. oxysporum*.

**Table 1:** *Fusarium* isolates used for pathogenicity testing and/or molecular characterization in this study.

*Fusarium* species	Isolate code	Location	Origin	Source*	Year isolated
Isolates used for pathogenicity testing and/or molecular characterization					
*F. oxysporum*	A13	Bedfordshire, UK, site 1	Onion bulb	V. Vagany, WCC	2009
*F. oxysporum*	A23	Bedfordshire, UK, site 2	Onion bulb	V. Vagany, WCC	2009
*F. oxysporum*	A28	Bedfordshire, UK, site 2	Onion bulb	V. Vagany, WCC	2009
*F. oxysporum*	A35	Bedfordshire, UK, site 3	Onion bulb	V. Vagany, WCC	2009
*F. oxysporum*	F1	Bedfordshire, UK, site 4	Onion bulb	V. Vagany, WCC	2010
*F. oxysporum*	195	Suffolk, UK, site 1	Onion bulb	C. Handy, WCC	2012
*F. oxysporum*	224	Suffolk, UK, site 1	Onion bulb	C. Handy, WCC	2012
*F. oxysporum*	244	Suffolk, UK, site 1	Onion bulb	C. Handy, WCC	2012
*F. oxysporum*	A21	Suffolk, UK, site 2	Onion bulb	V. Vagany, WCC	2009
*F. oxysporum*	R3	Suffolk, UK, site 3	Onion bulb	V. Vagany, WCC	2009
*F. oxysporum*	M1	Suffolk, UK, site 4	Onion bulb	V. Vagany, WCC	2010
*F. oxysporum*	M9	Suffolk, UK, site 4	Onion bulb	V. Vagany, WCC	2010
*F. oxysporum*	G12	Suffolk, UK, site 5	Onion bulb	V. Vagany, WCC	2009
*F. oxysporum*	K3b	Suffolk, UK, site 6	Onion bulb	V. Vagany, WCC	2009
*F. oxysporum*	S1B	Essex, UK, site 1	Onion bulb	V. Vagany, WCC	2009
*F. oxysporum*	A14	Essex, UK, site 2	Onion bulb	V. Vagany, WCC	2009
*F. oxysporum*	A19	Essex, UK, site 2	Onion bulb	V. Vagany, WCC	2009
*F. oxysporum*	NL70/7	Essex, UK, site 3	Onion bulb	V. Vagany, WCC	2010
*F. oxysporum*	A1_2	Warwickshire, UK	Onion bulb	V. Vagany, WCC	2008
*F. oxysporum* f. sp. *cepae*	FUS2	Lincolnshire, UK	Onion bulb	R. Noble, East Malling Research	Unknown
*F. oxysporum*	55	Lincolnshire, UK, site 1	Onion bulb	C. Handy, WCC	2012
*F. oxysporum*	84	Lincolnshire, UK, site 1	Onion bulb	C. Handy, WCC	2012
*F. oxysporum*	125	Lincolnshire, UK, site 1	Onion bulb	C. Handy, WCC	2012
*F. oxysporum*	RO2	Lincolnshire, UK, site 2	Onion bulb	V. Vagany, WCC	2010
*F. oxysporum*	FUS1	Nottinghamshire, UK	Onion bulb	R. Noble, East Malling Research	Unknown
*F. oxysporum*	FUS3	Nottinghamshire, UK	Onion bulb	R. Noble, East Malling Research	Unknown
*F. oxysporum*	PG	Cambridgeshire, UK	Onion bulb	T. O'Neill, ADAS	Unknown
*F. oxysporum*	CB3	UK	Onion set	C. Handy, WCC	2012
*F. oxysporum*	HB17	UK	Onion set	C. Handy, WCC	2012
*F. oxysporum*	HB6	UK	Onion set	C. Handy, WCC	2012
*F. oxysporum*	JB4	UK	Onion set	C. Handy, WCC	2012
*F. oxysporum*	NRRL 54002 (FO47)	France	Soil	ARS collection	Unknown
*F. oxysporum*	HAZ	USA	Onion bulb	H. van den Biggelaar, Hazera seeds	Unknown
*F. oxysporum*	L2‐1	UK, site 1	Leek	A. Taylor, WCC	2011
*F. oxysporum*	L9‐1	UK, site 2	Leek	A. Taylor, WCC	2011
*F. oxysporum*	ATCC90245	Colorado, USA	Pinto bean	ATCC collection	1990
*F. oxysporum* f. sp. *pisi* race 1	FOP1	UK	Pea	C. Linfield, WCC	Unknown
*F. oxysporum* f. sp. *pisi* race 2	FOP2	UK	Pea	C. Linfield, WCC	Unknown
*F. oxysporum* f. sp. *pisi* race 5	FOP5	UK	Pea	C. Linfield, WCC	Unknown
*F. oxysporum* f. sp. *pisi*	NRRL36311	The Netherlands	Pea	ARS collection	Unknown
*F. oxysporum* f. sp. *lini*	FOLIN	UK	Linseed	C. Linfield, WCC	2010
*F. oxysporum* f. sp. *dianthi*	R207	UK	Carnation	C. Linfield, WCC	Unknown
*F. oxysporum* f. sp. *narcissi*	FOXN7	UK	Daffodil	C. Handy, WCC	2013
*F. oxysporum* f. sp. *narcissi*	FOXN139	UK	Daffodil	C. Handy, WCC	2013
*F. oxysporum* f. sp. *freesia*	NRRL26990	The Netherlands	Freesia	ARS collection	Unknown
*F. oxysporum* f. sp. *freesia*	NRRL26988	The Netherlands	Freesia	ARS collection	Unknown
*F. oxysporum* f. sp. *cubense*	E421A3	UK	Banana	C. Nellist, WCC	Unknown
*F. avanaceum*	L5	UK, site 1	Leek	A. Taylor, WCC	2011
*F. proliferatum*	A8	Bedfordshire, UK, site 3	Onion bulb	V. Vagany, WCC	2009
*F. proliferatum*	A40	Bedfordshire, UK, site 3	Onion bulb	V. Vagany, WCC	2009
*F. proliferatum*	SP1‐2	Spain	Onion bulb	V. Vagany, WCC	2010
*F. redolens*	NL96	Essex, UK, site 3	Onion bulb	V. Vagany, WCC	2010
*F. oxysporum* f. sp. *lycopersici* race 3	NRRL54003 (MN25)	USA	Tomato	ARS Collection	Unknown
Genome sequenced isolates used for comparison in molecular characterization					
*F. oxysporum* f. sp. *pisi*	NRRL37622 (HDV247)	Unknown	Pea	ARS Collection	Unknown
*F. oxysporum*	NRRL32931 (FOSC 3‐a)	USA	Human	ARS Collection	Unknown
*F. oxysporum* f. sp. *conglutinans*	NRRL54008 (PHW808)	USA	Brassica	ARS Collection	Unknown
*F. oxysporum* f. sp. *raphani*	NRRL54005 (PHW815)	France	Radish	ARS Collection	Unknown
*F. oxysporum* f. sp. *radicis‐lycopersici*	NRRL26381 (CL57)	USA	Tomato	ARS Collection	Unknown
*F. oxysporum* f. sp. *cubense*	NRRL54006 (II5)	Indonesia	Banana	ARS Collection	Unknown
*F. oxysporum* f. sp. *melonis*	NRRL26406	USA	Melon	ARS Collection	Unknown
*F. oxysporum* f. sp. *vasinfectum*	NRRL25433	China	Cotton	ARS Collection	Unknown
*F. oxysporum* f. sp. *lycopersici* race 2	NRRL34936 (FOL4287)	USA?	Tomato	ARS Collection	Unknown
*F. oxysporum*	Fo5176	Australia	*Brassica oleracea*	ARS Collection	Unknown

*ATCC, American Type Culture Collection, USA; ARS, Agricultural Research Service culture collection, USA; WCC, Warwick Crop Centre, University of Warwick, UK.

**Figure 2:**
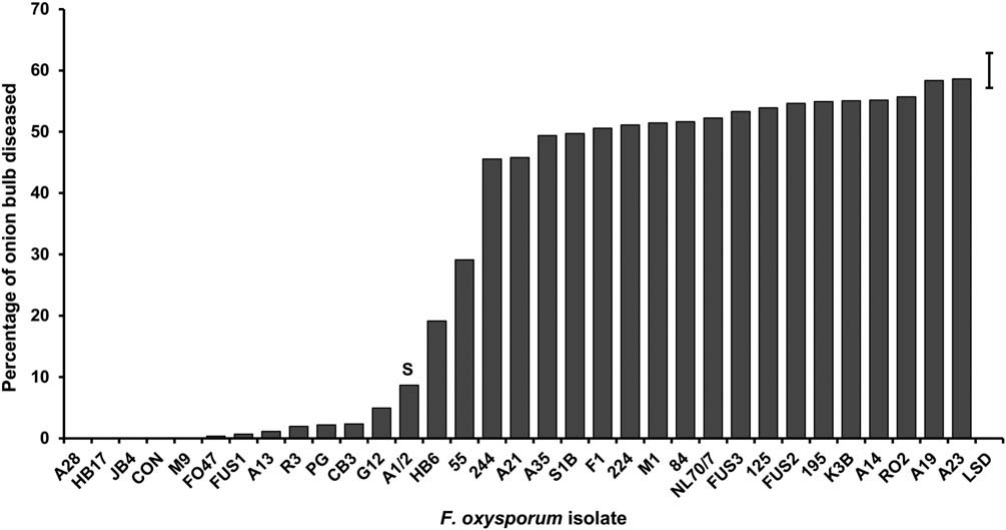
Pathogenicity of 32 *Fusarium oxysporum* isolates on onion bulbs (cv. Napoleon). Data shown are the percentage bulb areas diseased on bisected bulbs after 9 weeks at 20 °C. Error bar represents the least significant difference (LSD) (5%) level. An ‘S’ indicates the value above which there is a significant difference from the uninoculated control bulbs.

In the onion bulb test (cv. Napoleon), significant differences were observed in disease levels amongst the 32 *F. oxysporum* isolates (*P* < 0.001, Fig. 2), with 21 pathogenic isolates resulting in 8.7%–58.6% bulb area affected compared with the control (0%), and 11 non‐pathogenic isolates (including Fo47) having no significant effect (0%–5.0% bulb area affected). Of the 21 pathogenic isolates, A1_2 (8.7%) and HB6 (19.1%) showed lower levels of pathogenicity, whereas isolate 55 showed an intermediate level of pathogenicity (29.1%). Highly significant correlations were observed between *F. oxysporum* isolate pathogenicity in the bulb test and the seedling tests with cv. Napoleon (*r* = −0.91, *P* = 0.001) and cv. HZS (*r* = −0.88, *P* = 0.001). Pathogenic isolates resulting in significantly greater disease levels compared with the uninoculated controls in onion seedling or bulb tests were considered to be FOC.

### Molecular characterization: housekeeping genes

**Figure 3:**
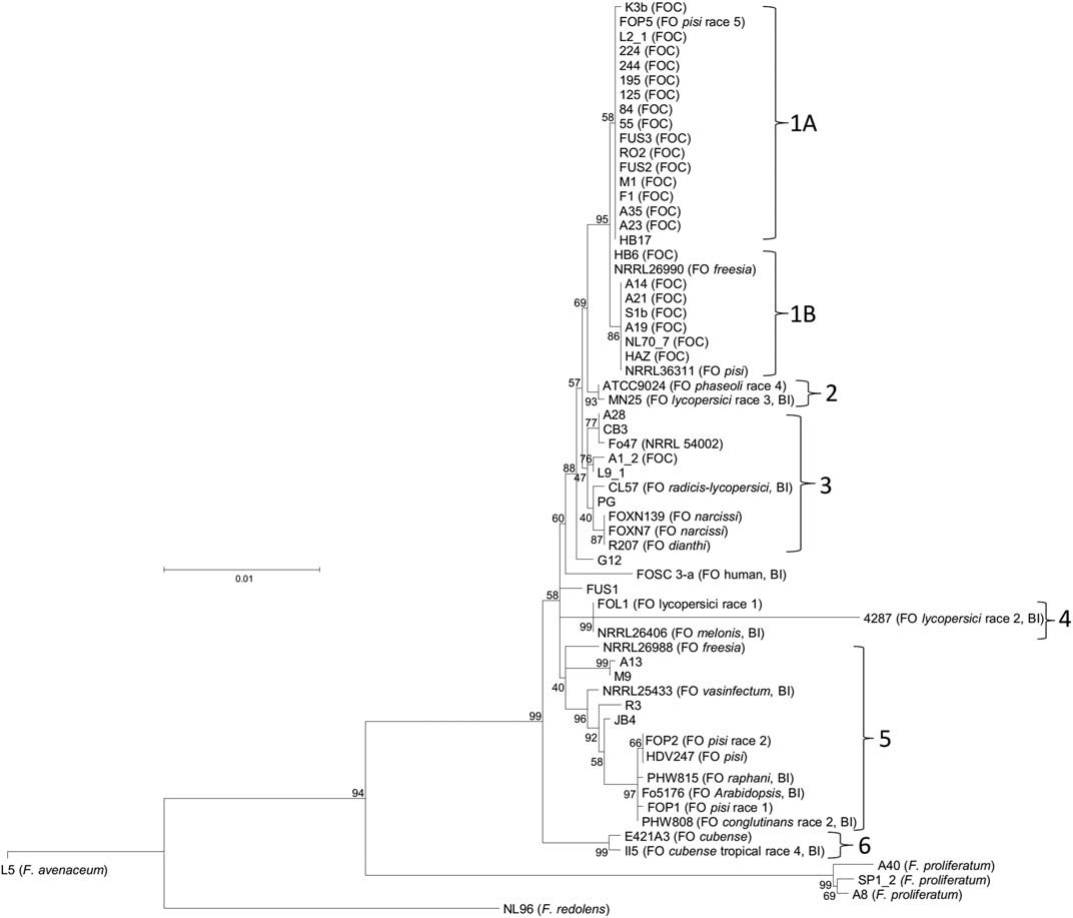
Maximum likelihood tree of *Fusarium* isolates from onion and other hosts based on a concatenated alignment of *translation elongation factor 1α* (*EF‐1α*) (GenBank accession numbers KP964857–KP964909), *RNA polymerase II second largest subunit* (*RPB2*) (GenBank accession numbers KP964804–KP964856) and *β‐tubulin* (*TUB2*) (GenBank accession numbers KP964910–KP964962) genes. Numbers represent bootstrap values from 1000 replicates. Scale bar indicates 0.01 substitutions per site. The tree is rooted through L5 (*F. avenaceum*) and this branch has been collapsed because of its distance from *F. oxysporum*. BI refers to a sequence derived from the genomes on the Broad Institute *Fusarium* database (Broad Institute/MIT, 2007).

The concatenated tree for *EF‐1α, RNA polymerase II second largest subunit* (*RPB2*) and *β‐tubulin* (*TUB2*) sequences resulted in the majority of the 53 *F. oxysporum* isolates being separated into six clades (Fig. 3). Isolates from onion were represented in clades 1, 3 and 5, and clade 1 contained all those that showed some level of pathogenicity in either the onion seedling or bulb tests (or both), with the exception of A1_2. Clade 1 also included isolate L2‐1 from infected leeks, which has also been shown to be pathogenic on onion bulbs (A. Taylor, A. Jackson & J. P. Clarkson, unpublished data). However, clade 1 also included the non‐pathogenic isolate HB17 (from onion sets) and isolates of *F. oxysporum* f. sp. *pisi* race 5 (FOP5), f. sp. *freesia* (NRRL26990) and f. sp. *pisi* (NRRL36311). Two non‐pathogenic onion isolates, A28 and CB3, showed high similarity to Fo47 in clade 3, whereas four other non‐pathogenic onion isolates (A13, M9, R3 and JB4) were placed in clade 5, together with several other f. spp. *Fusarium oxysporum* f. sp. *cubense* isolates were all placed in a separate and distinct clade (clade 6) and the other *Fusarium* species (*F. proliferatum*, *F. redolens* and *F. avenaceum*) formed distinct outgroups. Additional trees for each of the housekeeping genes were constructed using neighbour‐joining, minimum evolution and UPGMA (Unweighted Pair Group Method with Arithmetic Mean) methods, and similar topography was observed (data not shown).

### Molecular characterization: *SIX* genes

#### Fusarium oxysporum isolates from onion

Using primers based on the FOL or FOC genomes, homologues of *SIX3*, *SIX5*, *SIX7*, *SIX10*, *SIX12* and *SIX9*, *SIX14*, respectively were identified within the 31 *F. oxysporum* isolates from UK onions (Table 2) and USA isolate HAZ. All seven of these *SIX* genes were present in the 18 highly pathogenic isolates identified from the onion seedling and bulb tests (Table 2). In contrast, all *SIX* genes were absent in the 11 non‐pathogenic isolates, with the exception of isolate PG which contained *SIX9* (Table 2). Isolate 55, which had an intermediate level of pathogenicity on onion bulbs, and was mildly pathogenic on seedlings (cv. Napoleon), contained only *SIX9* and *SIX14*, whereas isolates HB6 and A1_2, which were weakly pathogenic on Napoleon bulbs (and on seedlings for A1_2), contained no *SIX* genes. All seven of the *SIX* genes identified were associated with a predicted signal peptide (Table 3).

**Table 2:** Presence/absence of *Secreted In Xylem* (*SIX*)*1–14* and three putative novel effectors in *Fusarium oxysporum* and other selected species.

*Fusarium* species	Host	Isolate code	Pathogenicity*	*SIX* genes†														*C5* ‡	*CRX1* §	*CRX2* ¶
				*1*	*2*	*3*	*4*	*5*	*6*	*7*	*8*	*9*	*10*	*11*	*12*	*13*	*14*			
*F. oxysporum* (FOC)	Onion	A23	B/S1/S2	−	−	+	−	+	−	+	−	+	+	−	+	−	+	+	+	1
*F. oxysporum* (FOC)	Onion	A19	B/S1/S2	−	−	+	−	+	−	+	−	+	+	−	+	−	+	+	+	−
*F. oxysporum* (FOC)	Onion	RO2	B/S1/S2	−	−	+	−	+	−	+	−	+	+	−	+	−	+	+	+	1
*F. oxysporum* (FOC)	Onion	A14	B/S1/S2	−	−	+	−	+	−	+	−	+	+	−	+	−	+	+	+	−
*F. oxysporum* (FOC)	Onion	K3B	B/S1/S2	−	−	+	−	+	−	+	−	+	+	−	+	−	+	+	+	1
*F. oxysporum* (FOC)	Onion	195	B/S1/S2	−	−	+	−	+	−	+	−	+	+	−	+	−	+	+	+	1
*F. oxysporum* (FOC)	Onion	FUS2	B/S1/S2	−	−	+	−	+	−	+	−	+	+	−	+	−	+	+	+	1
*F. oxysporum* (FOC)	Onion	125	B/S1/S2	−	−	+	−	+	−	+	−	+	+	−	+	−	+	+	+	1
*F. oxysporum* (FOC)	Onion	FUS3	B/S1/S2	−	−	+	−	+	−	+	−	+	+	−	+	−	+	+	+	1
*F. oxysporum* (FOC)	Onion	NL70/7	B/S1/S2	−	−	+	−	+	−	+	−	+	+	−	+	−	+	+	+	−
*F. oxysporum* (FOC)	Onion	84	B/S1/S2	−	−	+	−	+	−	+	−	+	+	−	+	−	+	+	+	1
*F. oxysporum* (FOC)	Onion	M1	B/S1/S2	−	−	+	−	+	−	+	−	+	+	−	+	−	+	+	+	1
*F. oxysporum* (FOC)	Onion	224	B/S1/S2	−	−	+	−	+	−	+	−	+	+	−	+	−	+	+	+	1
*F. oxysporum* (FOC)	Onion	F1	B/S1/S2	−	−	+	−	+	−	+	−	+	+	−	+	−	+	+	+	1
*F. oxysporum* (FOC)	Onion	S1B	B/S1/S2	−	−	+	−	+	−	+	−	+	+	−	+	−	+	+	+	−
*F. oxysporum* (FOC)	Onion	A35	B/S1/S2	−	−	+	−	+	−	+	−	+	+	−	+	−	+	+	+	1
*F. oxysporum* (FOC)	Onion	A21	B/S1/S2	−	−	+	−	+	−	+	−	+	+	−	+	−	+	+	+	−
*F. oxysporum* (FOC)	Onion	244	B/S1/S2	−	−	+	−	+	−	+	−	+	+	−	+	−	+	+	+	1
*F. oxysporum* (FOC)	Onion	55	B/S1	−	−	−	−	−	−	−	−	+	−	−	−	−	+	+	+	1
*F. oxysporum* (FOC)	Onion	HB6	B	−	−	−	−	−	−	−	−	−	−	−	−	−	−	−	−	−
*F. oxysporum* (FOC)	Onion	A1_2	B/S1	−	−	−	−	−	−	−	−	−	−	−	−	−	−	−	−	−
*F. oxysporum*	Onion	G12	−	−	−	−	−	−	−	−	−	−	−	−	−	−	−	−	−	2
*F. oxysporum*	Onion	CB3	−	−	−	−	−	−	−	−	−	−	−	−	−	−	−	−	−	−
*F. oxysporum*	Onion	PG	−	−	−	−	−	−	−	−	−	+	−	−	−	−	−	−	−	3
*F. oxysporum*	Onion	R3	−	−	−	−	−	−	−	−	−	−	−	−	−	−	−	−	−	−
*F. oxysporum*	Onion	A13	−	−	−	−	−	−	−	−	−	−	−	−	−	−	−	−	−	−
*F. oxysporum*	Onion	FUS1	−	−	−	−	−	−	−	−	−	−	−	−	−	−	−	−	−	1
*F. oxysporum*	Onion	M9	−	−	−	−	−	−	−	−	−	−	−	−	−	−	−	−	−	−
*F. oxysporum*	Onion	JB4	−	−	−	−	−	−	−	−	−	−	−	−	−	−	−	−	−	−
*F. oxysporum*	Onion	HB17	−	−	−	−	−	−	−	−	−	−	−	−	−	−	−	−	−	−
*F. oxysporum*	Onion	A28	−	−	−	−	−	−	−	−	−	−	−	−	−	−	−	−	+	2
*F. oxysporum*	–	FO47	−	−	−	−	−	−	−	−	−	−	−	−	−	−	−	−	−	−
*F. oxysporum*	Onion	HAZ	(+)	−	−	+	−	+	−	+	−	+	+	−	+	−	+	+	+	+
*F. oxysporum*	Leek	L2‐1	(+)	−	−	+	−	+	−	+	−	+	+	−	+	−	+	+	+	+
*F. oxysporum*	Leek	L9‐1	(−)	−	−	−	−	−	−	−	−	−	−	−	−	−	−	−	−	−
																				
*F. oxysporum* f. sp. *lycopersici*	Tomato (race 3)	NRRL54003 (MN25)	NT	+	+	+	−	+	+	+	+	+	+	+	+	+	+	−	−	−
*F. oxysporum* f. sp. *lycopersici*	Tomato (race 1)	FOL1	NT	NT	NT	NT	+	NT	NT	NT	NT	NT	NT	NT	NT	NT	NT	−	−	−
*F. oxysporum* f. sp. *phaseoli*	Pinto bean (race 4)	ATCC90245	NT	−	−	−	−	−	+	−	+	−	−	+	−	−	−	−	−	−
*F. oxysporum* f. sp. *pisi*	Pea (race 1)	FOP1	NT	−	−	−	−	−	−	+	−	−	+	+	+	−	+	−	−	−
*F. oxysporum* f. sp. *pisi*	Pea (race 2)	FOP2	NT	−	−	−	−	−	−	−	−	−	−	−	−	+	+	−	−	+
*F. oxysporum* f. sp. *pisi*	Pea (race 5)	FOP5	NT	−	−	−	−	−	−	−	−	−	−	−	−	+	−	−	−	−
*F. oxysporum* f. sp. *pisi*	Pea	NRRL36311	NT	−	−	−	−	−	−	−	−	−	−	−	−	−	+	−	−	−
*F. oxysporum* f. sp. *lini*	Linseed	FOLIN	NT	−	−	−	−	−	−	+	−	−	+	−	+	+	−	−	−	−
*F. oxysporum* f. sp. *dianthi*	Carnation	R207	NT	−	−	−	−	−	−	+	−	+	+	−	+	−	−	−	−	−
*F. oxysporum* f. sp*. narcissi*	Daffodil	FOXN7	NT	−	−	−	−	−	−	+	−	+	+	−	+	−	−	−	−	−
*F. oxysporum* f. sp*. narcissi*	Daffodil	FOXN139	NT	−	−	−	−	−	−	+	−	+	+	−	+	−	−	−	−	−
*F. oxysporum* f. sp. *freesia*	Freesia	NRRL26990	NT	−	−	−	−	−	−	−	−	−	−	−	−	−	−	−	−	−
*F. oxysporum* f. sp. *freesia*	Freesia	NRRL26988	NT	−	−	−	−	−	−	+	−	−	+	−	+	+	+	−	−	−
*F. oxysporum* f. sp. *cubense*	Banana	E421A	NT	+	−	−	−	−	−	−	+	−	−	−	−	+	−	−	−	−
*F. proliferatum*	Onion	A8	(+)	−	−	−	−	−	−	−	−	−	−	−	−	−	−	−	−	+
*F. proliferatum*	Onion	A40	(+)	−	−	−	−	−	−	−	−	−	−	−	−	−	−	−	−	+
*F. proliferatum*	Onion	SP1‐2	(+)	−	−	−	−	−	−	−	−	−	−	−	−	−	−	−	−	+
*F. avenaceum*	Leek	L5	NT	−	−	−	−	−	−	−	−	−	−	−	−	−	−	−	−	−
*F. redolens*	Onion	NL96	(−)	−	−	−	−	−	−	−	−	−	−	−	−	−	−	−	−	−

*Isolate pathogenicity: B, pathogenic on onion bulbs; S1, pathogenic on onion seedlings cv. Napoleon; S2, pathogenic on onion seedlings cv. HZS; –, non‐pathogenic; NT, not tested. Symbols in parentheses refer to preliminary, unpublished pathogenicity data on onion bulbs and/or seedlings.

^†^GenBank accession numbers KP964963–KP965006.

^‡^GenBank accession number KP965007.

^§^GenBank accession number KP965011.

^¶^GenBank accession numbers KP965008–KP965010 and KP965012–KP965017. Numbers indicate sequence type (*Fusarium* isolates from onion only, Fig. 6); +, presence of CRX2; −, absence of CRX2.

**Table 3:** Putative effector genes in *Fusarium oxysporum* f. sp. *cepae* with associated nucleotide and protein percentage identities using blast (Boratyn *et al*., 2013) in comparison with *F. oxysporum* f. sp. *lycopersici* unless otherwise stated.

Gene	Nucleotide ID	Protein ID	Signal peptide
*SIX3*	91	86	Yes
*SIX5*	90	73	Yes
*SIX7*	91	82	Yes
*SIX9*	90*	82*	Yes
*SIX10*	96†	91†	Yes
*SIX12*	95	94	Yes
*SIX14*	62	78	Yes
*C5*	No homology	No homology	No
*CRX1*	89‡	80‡	Yes
*CRX2*	100‡	93‡	Yes

*Percentage identity to *SIX9a* in an *Arabidopsis*‐infecting *F. oxysporum* isolate (HQ260603).

^†^Percentage identity does not include an intron which is present in *F. oxysporum* f. sp. *cepae*, but not in *F. oxysporum* f. sp. *lycopersici*.

^‡^Closest match *F. oxysporum* CL57: FOCG_17596.1: hypothetical protein.

The *SIX3*, *SIX5*, *SIX7*, *SIX10* and *SIX12* sequences from FOC all had a high level of homology with the corresponding FOL *SIX* genes, ranging from 85% to 96% nucleotide identity, whereas the FOC *SIX9* and *SIX14* homologues were more divergent (73% and 62% nucleotide identity, respectively; Table 3). The FOC *SIX9* gene also showed very high homology (90% nucleotide identity) with *SIX9a* identified in the *F. oxysporum* isolate infecting *Arabidopsis* and brassica (Thatcher *et al*., 2012). Sequences for *SIX3*, *SIX7*, *SIX10*, *SIX12* and *SIX14* were identical across all the FOC isolates (where present), including isolate HAZ from the USA. In addition, the FOC *SIX3* gene identified in our study had a 100% match to the *SIX3* sequence from a Japanese FOC isolate (Genbank accession number BAP74165). No other *SIX* gene homologues were identified in the FUS2 genome, confirming the negative polymerase chain reaction (PCR) results.

#### Fusarium oxysporum from different hosts and other Fusarium species from onion/leek

Two *F. oxysporum* isolates from leek were included in this study, one of which was pathogenic on both onion and leek (L2‐1; A. Taylor, A. Jackson and J. P. Clarkson, unpublished data). This isolate had an identical *SIX* gene profile to the isolates which were pathogenic on onion and identical sequences. Five *SIX* genes were identified in *F. oxysporum* f. spp. *pisi* race 1 (FOP1; *SIX7*, *SIX10*, *SIX11*, *SIX12* and *SIX14*) and *freesia* (NRRL26988; *SIX7*, *SIX10*, *SIX12*, *SIX13* and *SIX14*). However, a second *F. oxysporum* f. sp. *freesia* isolate (NRRL26990) had no *SIX* genes at all. The *SIX1* gene was only identified in *F. oxysporum* f. sp. *cubense* and FOL isolates, whereas *SIX2* and *SIX4* were unique to FOL. *SIX3*, *SIX5* and *SIX12* were only found in FOC and FOL. *SIX6* was only detected in FOL and f. sp. *phaseoli*, whereas *SIX7* and *SIX10* were found in FOC, FOL and f. spp. *pisi* race 1, *dianthi*, *narcissi* and *freesia*. *SIX8* was detected in FOL and *F. oxysporum* f. sp. *cubense* and f. sp. *phaseoli*, whereas *SIX9* was detected in FOL using the FOL primers, and in FOC, *F. oxysporum* f. sp. *dianthi* and f. sp. *narcissi* using the FOC primers. *SIX11* was found in *F. oxysporum* f. sp. *phaseoli*, f. sp. *pisi* race 1 and FOL. *SIX13* was detected in *F. oxysporum* f. sp. *pisi* races 2 and 5, f. sp. *dianthi*, f. sp. *narcissi*, f. sp. *freesia* and FOL. *SIX14* was detected in FOC, FOL, *F. oxysporum* f. sp. *pisi* and f. sp. *freesia*. None of the other *Fusarium* species tested contained any of the *SIX* genes.

**Figure 4:**
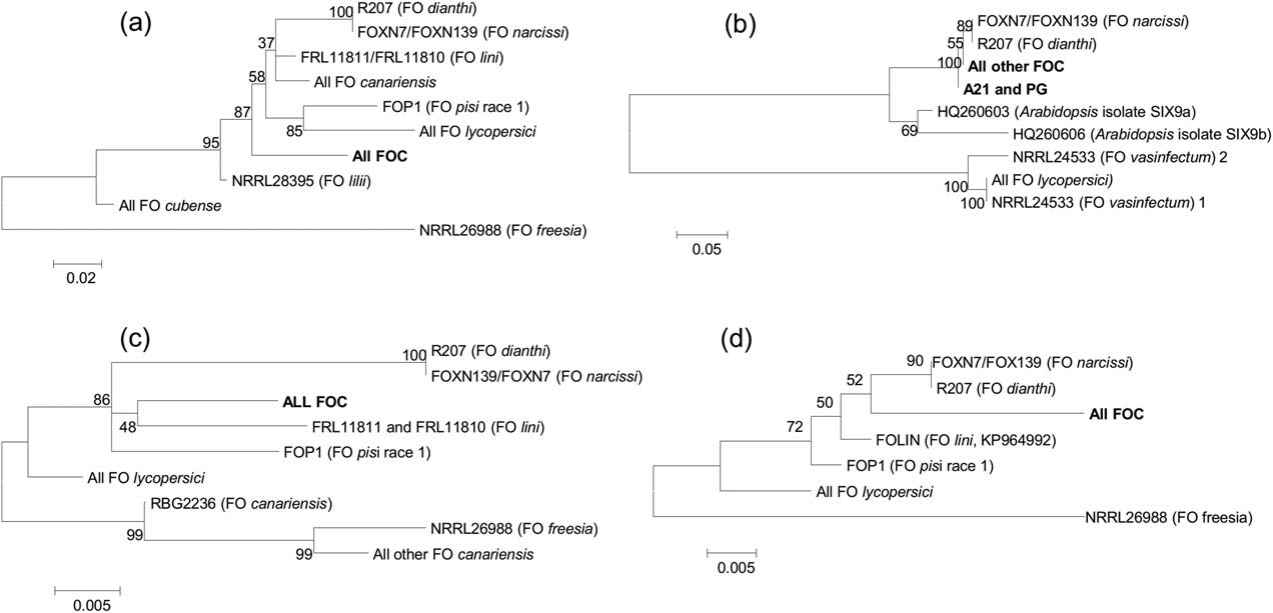
Maximum likelihood trees of *Fusarium* isolates from onion and other hosts based on (a) *SIX 7*, (b) *SIX9*, (c) *SIX10*, and (d) *SIX12* gene sequences. Numbers represent bootstrap values from 1000 replicates. Scale bars indicate the number of substitutions per site. All FO *lycopersici* refers to the genome sequenced isolates listed in Table 1 as well as additional identical sequences obtained from a BLAST search. All FO *cubense* refers to the genome sequenced isolate II5 as well as identical BLAST hits. Sequences of other NRRL isolates were extracted from genome sequences (Broad Institute/MIT, 2007). All FO *canariensis* and FO *lini* isolates (with the exception of FOLIN, SIX12) are as described by Laurence *et al*. (2015).

*SIX* gene sequence variation was observed across the different *F. oxysporum* f. spp., and phylogenetic trees showed that FOC isolates were clearly separated from the other f. spp. based on *SIX7*, *SIX10* and *SIX12*, but not *SIX9* (Fig. 4). The FOL *SIX9* gene formed part of a separate clade to the *SIX9a* and *SIX9b* genes identified in the *Arabidopsis/*brassica‐infecting isolate (Fo5176), whereas the *SIX9* sequence from FOC was in the same clade as SIX9a/SIX9b and was closer to *SIX9a* than *SIX9b*. The separation of FOC isolates from other *F. oxysporum* f. spp. was less clear for *SIX9* as the sequence was very similar to that from both *F. oxysporum* f. spp. *narcissi* and *dianthi*. *Fusarium oxysporum* isolates from carnation and *Narcissus* could not be distinguished on the basis of any of the *SIX* gene sequences. For *SIX5*, six FOC isolates (S1b, A14, A19, A21, NL70/7 and HAZ) had a single base change at position 316 (G to A, Fig. S1, see Supporting Information) causing a single amino acid change from R to K. All of these isolates were in clade 1b in the housekeeping gene tree (Fig. 3). For *SIX9*, two onion isolates, A21 (pathogenic) and PG (non‐pathogenic), had a different sequence type, differing by a single base pair (T instead of A), resulting in an amino acid change from D to V.

### Molecular characterization: putative novel effectors

Two putative novel effectors, *C5* and *CRX1*, were detected in all of the 18 FOC isolates that were highly pathogenic across both seedling and bulb assays (Table 2), although *CRX1* was also found in isolate A28 which was non‐pathogenic. The presence of a third putative effector (*CRX2*) was partially associated with pathogenicity, being detected in 13 of the 18 highly pathogenic isolates (Table 2) and in four non‐pathogenic isolates (A28, FUS1, G12 and PG). FOC isolate 55, which demonstrated ‘intermediate’ pathogenicity on onion bulbs, contained *C5*, *CRX1* and *CRX2* (as well as *SIX9* and *SIX14*), whereas FOC isolate A1_2, which was weakly pathogenic, had no putative effectors. *C5* and *CRX1* were not present in any of the other *F. oxysporum* f. spp., whereas *CRX2* was detected in f. sp*. pisi* race 2, *F. redolens* and all the *F. proliferatum* isolates. Genes with homology to *CRX1/CRX2* were also found in the genome sequences of *F. oxysporum* f. sp. *pisi* (HDV247: FOVG_18080) and f. sp. *radicis‐lycopersici* (CL57: FOCG_17596 and FOCG_16735) (Broad Institute/MIT, 2007). *C5* was not present in any of the published *Fusarium* genomes (Table 1) and there are no sequence matches at the National Center for Biotechnology Information (NCBI).

**Figure 5:**
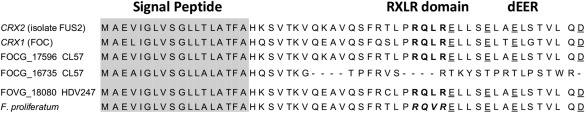
Amino acid alignment of putative RxLR effectors from *Fusarium oxysporum* and *F. proliferatum*. The signal peptide (as predicted by SignalP) is shaded in light grey, whereas the RxLR domain is shown in bold (bold and italics for an incomplete RxLR domain). Amino acids that often occur after an RxLR domain (dEER) are underlined. Sequences from HDV247 and CL57 were obtained from the Broad Institute *Fusarium* database (Broad Institute/MIT, 2007).

**Figure 6:**
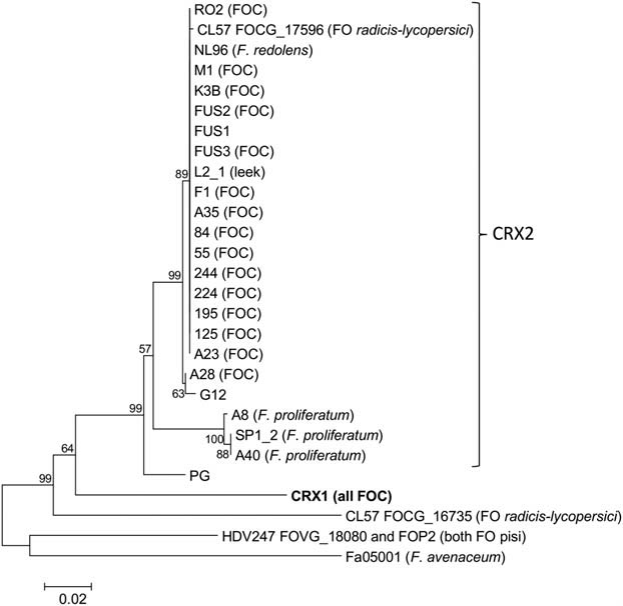
Neighbour‐joining tree of *Fusarium oxysporum CRX1* and *CRX2* genes and their homologues. Numbers represent bootstrap values from 1000 replicates. The scale bar indicates 0.02 substitutions per site. Sequences from HDV247 and CL57 were obtained from the Broad Institute *Fusarium* database (Broad Institute/MIT, 2007). The sequence from Fa05001 was obtained from an assembled genome (GenBank accession number GCA_000769215).

The putative effectors *CRX1* and *CRX2* in *F. oxysporum* contained RxLR domains close to the N‐terminus (at amino acid positions 37–40, Fig. 5), whereas *F. proliferatum* isolates had a modified (RQVR) sequence in this position. The RxLR domains were flanked by modified dEER domains, defined by the presence of >10% D or E residues (Jiang *et al*., 2008). All *C5* and *CRX1* sequences were identical across the *F. oxysporum* isolates and, based on the partial coding DNA sequence, a phylogenetic tree clearly separated *CRX1* and *CRX2* (Fig. 6). The majority of the 13 (pathogenic) FOC isolates that contained *CRX2* had identical sequences, although the non‐pathogenic isolate FUS1 also had the same sequence (Fig. 6). Three non‐pathogenic isolates from onion (A28, G12 and PG) had slightly different *CRX2* sequence types and, in the case of isolates PG and G12, this resulted in a stop codon in the middle of the coding region (data not shown). *CRX2* sequences from *F. oxysporum* f. sp. *radicis‐lycopersici* (FOCG_17596) and *F. redolens* (NL96) were very similar to the predominant FOC sequence, whereas the three *F. proliferatum* isolates, which also contained *CRX2*, formed a distinct clade.

### Expression of putative effectors

**Figure 7:**
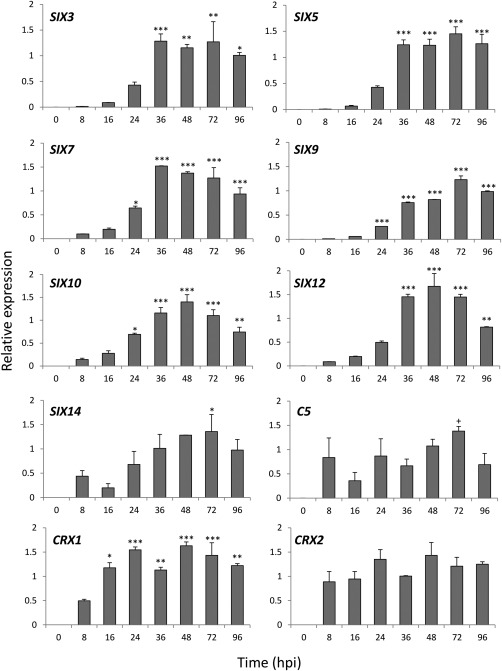
Quantitative expression of a set of putative effector genes in onion roots following inoculation with *Fusarium oxysporum* f. sp. *cepae* (FOC) isolate FUS2. Expression was calculated relative to *translation elongation factor 1α* (*EF‐1α*) and *β‐tubulin* (*TUB2*). Error bars show the standard error of the mean (SEM) of three replicates; hpi, hours post‐inoculation. Asterisks indicate expression levels significantly different from 8 hpi based on analysis of variance (ANOVA) followed by Tukey's test (*P < 0.05, **P < 0.01, ***P < 0.001). + indicates that the expression of *C5* was significantly higher at 72 hpi relative to 16 hpi (*P* < 0.05).

Using real‐time reverse transcription‐polymerase chain reaction (RT‐PCR), the seven SIX genes and the three putative effectors *C5*, *CRX1*, *CRX2* were all shown to be expressed *in planta* across the time course following inoculation with FOC isolate FUS2. *SIX3*, *SIX5*, *SIX7*, *SIX9*, *SIX10*, *SIX12* and *CRX1* showed significant increases in expression levels at 36–72 h post‐inoculation (hpi) compared with the first time point at 8 h (Fig. 7), whereas *SIX14* only showed a significant increase at 72 hpi. There was no increase in *C5* expression for any of the time points compared with 8 hpi, although a significant increase was detected between 16 and 72 hpi. *CRX2* expression levels did not change significantly across the time course.

## Discussion

In this study, we have demonstrated, for the first time, a clear association between the presence of seven *SIX* genes and two other putative effectors (*C5*, *CRX1*) in FOC and pathogenicity on onion. A third putative effector (*CRX2*) showed a partial association with pathogenicity. Pathogenicity tests on onion seedlings and bulbs were consistent across all the *F. oxysporum* isolates, and a strong correlation between pathogenicity results using two different onion cultivars in the seedling tests supports the suggestion that there is no cultivar × isolate interaction (Taylor *et al*., 2013).

Three of the seven *SIX* genes identified in FOC (*SIX3*, *SIX5* and *SIX7*) had been identified previously, but their presence was only related to pathogenicity on onion seedlings (Sasaki *et al*., 2015b), and not in a bulb test, which is more appropriate to disease expression in the field. In addition, this study did not provide evidence of expression *in planta*. The remaining four *SIX* genes (*SIX9*, *SIX10*, *SIX12* and *SIX14*) are reported here for the first time in FOC. The presence of *SIX* genes has similarly been associated with the pathogenicity of *F. oxysporum* isolates on tomato (*SIX1–7*; Lievens *et al*., 2009), cotton (*SIX6*; Chakrabarti *et al*., 2011) and banana (*SIX1*, *SIX7* and *SIX8*; Meldrum *et al*., 2012). The function of the 14 *SIX* genes detected so far in *F. oxysporum* is unclear as they show little or no homology with any known proteins (Fraser‐Smith *et al*., 2014). However, *SIX1*, *SIX3*, *SIX4*, *SIX5* and *SIX6* have all been shown to make a direct contribution to pathogenicity (Gawehns *et al*., 2014; Houterman *et al*., 2009; Ma *et al*., 2015; Rep, 2005; Takken and Rep, 2010; Thatcher *et al*., 2012).

One *F. oxysporum* isolate from leek (L2‐1) also shared the same effector gene profile as the pathogenic onion isolates. Preliminary work (Taylor A, Jackson A.C, Clarkson J.P, unpublished.) has demonstrated that this leek isolate is also pathogenic on onion bulbs, whereas another leek isolate (L9‐1), which was non‐pathogenic, lacked any of the effector genes. FOC has also been shown to cause basal rot in Welsh onion (*A. fistulosum*; Dissanayake *et al*., 2009a, 2009b; Sasaki *et al*., 2015b) and garlic (*A. sativum*; Rout *et al*., 2014), suggesting that FOC might be more appropriately named *F. oxysporum* f. sp. *allii*. However, it should be noted that, although FOC isolates from *A. fistulosum* can be pathogenic on *A. cepa* seedlings, they lack *SIX3*, *SIX5* and *SIX7* (Sasaki *et al*., 2015b), and the pathogenicity of these isolates on onion bulbs has yet to be tested.

It is likely that the FOC *SIX* genes, and possibly the other putative effectors, are located on dispensable/supernumerary chromosomes, as has been reported for FOL (Ma *et al*., 2010). FOC *SIX3* has been shown to be located on a small (4‐Mb) chromosome, which may be equivalent to a FOL supernumerary chromosome (Sasaki *et al*., 2015b). It has also been shown recently that *SIX3* and *SIX5* share the same promoter and appear to act as a gene pair, which is recognized by the *I‐2* resistance gene (Houterman *et al*., 2009; Ma *et al*., 2015). It has been suggested that this is unique to FOL, but our data and those of Sasaki *et al*. (2015a) confirm that these genes are also present in FOC. For all of the seven *SIX* genes in FOC, sequences were mostly identical and, similarly, there was also a high level of sequence conservation with those reported in FOL, suggesting a conservation of function. Indeed, five of the seven FOC *SIX* genes (*SIX3*, *SIX7*, *SIX10*, *SIX12* and *SIX14*) showed no intraspecific sequence differences within isolates originating from the UK, USA and Japan. This supports the suggestion that *SIX* genes in FOC have been acquired by horizontal gene transfer of the supernumerary chromosomes, as proposed for other *F. oxysporum* f. spp. (Fraser‐Smith *et al*., 2014; Laurence *et al*., 2015; Ma *et al*., 2010). A naturally occurring isolate of *F. oxysporum* from onion (isolate 55) identified in this study had only two of the seven *SIX* genes (*SIX9* and *SIX14*) and a corresponding intermediate level of pathogenicity. This phenomenon has not been observed so far in other f. spp., and may suggest a missing or mutated copy of a supernumerary chromosome.

In this study, we also report, for the first time, three novel putative effectors in FOC (*C5*, *CRX1* and *CRX2*), and the presence of *C5* and *CRX1* had an almost complete correlation with pathogenicity. *C5* has no homology to any sequenced gene, suggesting that it may be FOC specific, but both *CRX1* and *CRX2* contained RxLR domains, a motif found in many diverse oomycete candidate effector genes which, in some reports, has been shown to facilitate entry into host cells (Kale, 2012; Kale *et al*., 2010; Rehmany *et al*., 2005). Putative RxLR effectors have also been reported in FOL (Ma *et al*., 2013), but this is the first report of putative RxLR effectors in FOC. *CRX2* only had a partial association with pathogenicity in FOC, and also has very close homologues in *F. oxysporum* f. sp. *radicis‐lycopersici*, *F. proliferatum* and *F. redolens*. We also demonstrated, for the first time, that the *SIX* genes and the putative FOC‐specific effector *CRX1* are up‐regulated during the infection process, with levels of expression *in planta* increasing over a time course, further supporting the hypothesis that these genes play a role in pathogenicity. In addition, with the exception of *C5*, all other putative effectors and *SIX* genes had an associated signal peptide, supporting the hypothesis that they are secreted. However, *SIX12* in FOL, which is also reported to have no associated signal peptide, was found in the xylem sap of infected tomato plants, leading to the conclusion that it was secreted by a different mechanism (Schmidt *et al*., 2013). Additional functional analyses are now required to reveal the potential roles of the new putative effectors in pathogen virulence.

The characterization of all the *F. oxysporum* isolates using the three housekeeping genes *EF‐1α*, *RPB2* and *TUB2* showed that there was considerable genetic variation between isolates from onion, as reported previously using AFLP markers, *EF‐1α* and IGS sequencing (Galván *et al*., 2008; Sasaki *et al*., 2015b). Although all pathogenic isolates, with the exception of A1_2 (weakly pathogenic), were placed in clade 1, the same clade also contained a small number of non‐pathogenic isolates and some other f. spp., indicating that housekeeping genes are not useful for distinguishing between different *F. oxysporum* f. spp. or between pathogenic and non‐pathogenic isolates. However, one study found a partial association between IGS sequence and pathogenicity (Sasaki *et al*., 2015b), but it was not clear whether other *F. oxysporum* f. spp. share this sequence type. The non‐pathogenic *F. oxysporum* isolates from onion were generally scattered throughout the phylogenetic tree, suggesting that they are more diverse than the pathogenic isolates. Although all *F. oxysporum* isolates in this study were originally from diseased onion tissue, the isolation of non‐pathogenic isolates is common and they are likely to be either endophytes or saprophytes, which are secondary colonizers of infected roots or bulbs (Alabouvette *et al*., 2009).

Our main reason for selecting the additional *F. oxysporum* f. spp. for characterization in this study was to expand the knowledge concerning the distribution of *SIX* genes. A study of *SIX1–SIX7* in a wider range of *F. oxysporum* f. spp. suggested that only *SIX6* (in f. spp. *melonis* and *radicis‐cucumerinum*) and *SIX7* (in f. sp. *lilii*) were present in f. spp. other than FOL (Lievens *et al*., 2009). However, more recent work (and the release of whole genome sequences for selected *F. oxysporum* f. spp.) has shown that all of the 14 *SIX* genes are present in various complements in other f. spp. (Broad Institute/MIT, 2007; Fraser‐Smith *et al*., 2014; Laurence *et al*., 2015; Sasaki *et al*., 2015b). FOC isolates in this study contained seven of the 14 *SIX* genes, whereas five were identified for *F. oxysporum* f. sp. *freesia* (NRRL26988) and f. sp. *pisi* race 1. Interestingly, a second freesia isolate (NRRL26990) examined here contained no *SIX* genes at all, suggesting that it may not be pathogenic or may have been misidentified. We also found a range of *SIX* genes present in isolates of *F. oxysporum* f. sp. *pisi* and there appeared to be distinct variation between races in terms of *SIX* gene complement. The only previous literature on *SIX* genes in *F. oxysporum* f. sp. *pisi* showed an absence of *SIX6* in races 1, 2, 5 and 6 (Chakrabarti *et al*., 2011), which is in agreement with our findings.

In this study, an *F. oxysporum* f. sp. *dianthi* isolate (R207) contained *SIX7*, *SIX9*, *SIX10* and *SIX12*, which is in contrast with previous findings that reported an absence of *SIX1–7* in f. sp. *dianthi* isolates from the USA and the Netherlands (Lievens *et al*., 2009). Overall, our results, in combination with other reports, suggest that *SIX* gene complements and/or sequences can be used to separate *F. oxysporum* f. spp. without the need for pathogenicity testing. Recently, a quantitative PCR assay was developed for the detection of FOC *in planta* based on *SIX3* (Sasaki *et al*., 2015a). Although this test requires more rigorous validation against a range of *F. oxysporum* and other soil‐borne fungi, it could potentially be useful as a soil or plant test for FOC.

Finally, the other *Fusarium* species *F. proliferatum*, *F. avenaceum* and *F. redolens*, which were also isolated from diseased onions and leeks in this study, did not contain any *SIX* genes. This confirms the results for the isolates of *F. proliferatum* and *F. avenaceum* from sugar beet, where *SIX1* and *SIX6* were absent (Covey *et al*., 2014). This was also the case for *F. graminearum*, *F. solani* and *F. javanicum*, which lacked *SIX1*–*7* (Lievens *et al*., 2009). Whole genome sequences have recently become available for *F. avenaceum* isolates from barley and wheat (Lysøe *et al*., 2014), and these genomes were examined for the presence of all putative effectors. The only hit was for CRX2 (80% identity), which was only present in an isolate from barley (Fa05001, Fig. 6). The *F. proliferatum* and *F. avenaceum* isolates used in our study were also shown to be pathogenic on onion (A. Taylor, A. C. Jackson, and J. P. Clarkson, unpublished data) and must therefore possess a different mechanism of infection compared with FOC. Overall, our findings provide a greater understanding of pathogenicity in FOC, and could potentially improve the diagnosis and control of *Fusarium* basal rot of onion in the future.

## Experimental Procedures

### *Fusarium* isolates

*Fusarium* isolates were obtained from diseased onion roots and bulbs from different locations in the UK collected between 2008 and 2012 (Table 1), as described by Taylor *et al*. (2013). Thirty‐one *F. oxysporum* isolates were selected for subsequent molecular characterization and pathogenicity tests based on sample location, colony morphology, preliminary pathogenicity data (where available) and *EF‐1α* sequences of a few isolates (Taylor *et al*., 2013; Vágány, 2012), as well as the genome sequenced non‐pathogenic biocontrol agent Fo47 (NRRL54002). In addition, a genome‐sequenced isolate of FOL race 3 (MN25, NRRL54003), a FOC isolate from the USA (HAZ), two *F. oxysporum* isolates from diseased UK leeks (L2‐1, L9‐1) and 17 isolates of *F. oxysporum* f. spp. *pisi*, *dianthi*, *narcissi*, *cubense* and *lycopersici*, as well as three other *Fusarium* species from diseased onions/leeks (*F. avenaceum*, *F. proliferatum* and *F. redolens*), were also obtained from various researchers and culture collections for comparison in the molecular characterization studies (Table 1).

### Pathogenicity testing: seed inoculation

The 31 *F. oxysporum* isolates from UK onions and the non‐pathogenic isolate Fo47 were assessed for their pathogenicity on onion seedlings as described by Taylor *et al*. (2013). Two experiments were carried out: one using cv. Napoleon (Syngenta, Cambridge, Cambridgeshire UK) and the second using cv. HZS (a standard FOC susceptible line from Hazera Seeds, Made, The Netherlands). For each experiment, there were four independent replicates over time, each consisting of a tray of 28 onion seeds per isolate, which were positioned in a glasshouse using an alpha design (Genstat v.12, VSN International, Hemel Hempstead, Hertfordshire, UK). Three trays of uninoculated control treatments (seeds soaked in sterile distilled water (SDW)) were included in each replicate experiment. The number of surviving seedlings was recorded after 6 weeks and the percentage survival was calculated relative to germination to allow for any variation in germination between replicates. Significant differences between treatments (isolates) for these data were assessed using residual (or restricted) maximum likelihood (REML) analysis (Welham & Thompson, 1997) in GenStat. The Pearson product moment correlation coefficient was also calculated to determine the correlation between the two cultivars.

### Pathogenicity testing: bulb inoculation

The same 32 *F. oxysporum* isolates used in the seedling tests were assessed for pathogenicity on healthy, stored onion bulbs (cv. Napoleon). The outer scales of the bulbs were removed to leave a single brown layer of skin, after which the basal plate of each onion was cut off and the bulb surface was sterilized with 70% ethanol. A potato dextrose agar (PDA) plug (8 mm) taken from the edge of an actively growing colony of each *F. oxysporum* isolate (grown for 7 days at 20 °C) was then positioned on the basal plate of each bulb. Control bulbs were inoculated with a sterile plug of PDA. Bulbs were placed on moist tissue in a plastic box (four per box) inside a sealed plastic bag to maintain high humidity and incubated at 20 °C in the dark. Boxes were randomized in trays following an alpha design. After 48 h, each bulb was wrapped with cling‐film to ensure that the agar plug did not dry out. After 9 weeks, each bulb was bisected longitudinally and a digital image was taken (including a 10‐cm scale bar). Images were then analysed using ImageJ software (Schneider *et al*., 2012) to quantify the area of infection as a percentage of the total bulb area. Three independent replicates (four onion bulbs per replicate) were set up for each *F. oxysporum* isolate and significant differences between isolate disease area data were analysed using REML in GenStat. Pearson product moment correlation coefficients were calculated to determine correlations between disease data from seedling and bulb tests.

### Molecular characterization of *Fusarium* isolates: housekeeping genes

Molecular characterization through sequencing of housekeeping genes was carried out for the 32 *F. oxysporum* isolates used in the pathogenicity tests, as well as 21 other f. spp. of *F. oxysporum* and *Fusarium* species (Table 1). Each isolate was grown on PDA for 4–7 days at 25 °C, and three agar plugs (5 mm) taken from the leading edge were placed in a 50‐mL tube containing 25 mL of sterile 50% potato dextrose broth (PDB). After incubation at 25 °C for 5 days and centrifugation at 3000 × g for 5 min, excess liquid was removed and the mycelium was rinsed twice with SDW. Finally, excess water was removed and the mycelium was flash frozen in liquid nitrogen before lyophilizing for 24 h. DNA was then extracted from approximately 20 mg of mycelium using a DNeasy plant mini kit (Qiagen, Hilden, Germany) with minor modifications, whereby the mycelium was first homogenized in a lysing matrix A tube (MP Biomedicals, Santa Ana, CA, USA) placed in a FastPrep‐24™ machine (MP Biomedicals) set at 6 m/s for 40 s. The manufacturer's protocol was then followed with the addition of an extra centrifugation step (12 500 × g for 5 min) after the cell lysis stage. DNA integrity was confirmed by gel electrophoresis.

PCR amplification and sequencing of *EF‐1α*, *RPB2* and *TUB2* was carried out for all *Fusarium* isolates using published primers (Table 4) with reactions set up using REDTaq® ReadyMix® (Sigma‐Aldrich Gillingham, Dorset, UK) in 20‐µL volumes containing approximately 50 ng of DNA and a final concentration of 0.5 µm of each primer. For *EF‐1α*, the thermocycling conditions were as follows: one cycle of 5 min at 94 °C; 40 cycles of 45 s at 94 °C, 30 s at 64 °C and 2 min at 72 °C, followed by one cycle of 10 min at 72 °C. For *RPB2*, the conditions were as follows: one cycle of 1.5 min at 94 °C; 40 cycles of 30 s at 94 °C, 1.5 min at 60 °C and 2 min at 68 °C, followed by one cycle of 10 min at 68 °C. For *TUB2*, the conditions were as follows: one cycle of 3 min at 95 °C; 35 cycles of 1 min at 94 °C, 30 s at 60 °C and 1 min at 72 °C, followed by one cycle of 10 min at 72 °C. All PCR amplicons were purified using a QIAquick PCR Purification Kit (Qiagen), sequenced using forward and reverse primers, and contigs were constructed using the SeqBuilder package of DNASTAR® Lasergene® version 10 (DNASTAR Inc., Madison, WI, USA). Sequences were aligned (clustalw method, Thompson et al., 1994), concatenated using mega version 5.1 (Tamura *et al*., 2011) and a maximum likelihood tree was constructed using the calculated best model, Kimura‐2‐parameter plus gamma (Kimura, 1980). Bootstrap consensus trees were inferred from 1000 replicates (Felsenstein, 1985). Sequences from published *F. oxysporum* genomes (Broad Institute/MIT, 2007) were also included in this analysis and were identified using a Broad Institute (BI) label.

**Table 4:** Primer pairs used for molecular characterization of *Fusarium* isolates with product size, annealing temperature and relevant publications.

Gene*	Primers	Sequence 5′–3′ (forward primer/reverse primer)	Product size	Annealing temperature (ºC)	Publication
*TUB2*	T1/T22	AACATGCGTGAGATTGTAAGT/TCTGGATGTTGTTGGGAATCC	∼1500	60	O'Donnell and Cigelnik (1997)
*RPB2*	7cF/11aR	ATGGGYAARCAAGCYATGGG/GCRTGGATCTTRTCRTCSACC	881	57	O'Donnell *et al*. (2007)
*EF‐1α*	exTEF‐F/FUexTEF‐R	ACCCGGTTCAAGCATCCGATCTGCGA/AGCTTGCCRGACTTGATCTCACGCTC	1269	64	Vágány (2012)
*SIX1*	SIX1	GTATCCCTCCGGATTTTGAGC/AATAGAGCCTGCAAAGCATG	992	59	Lievens *et al*. (2009)
*SIX2*	SIX2	CAACGCCGTTTGAATAAGCA/TCTATCCGCTTTCTTCTCTC	749	59	Lievens *et al*. (2009)
*SIX3*	SIX3	CCAGCCAGAAGGCCAGTTT/GGCAATTAACCACTCTGCC	608	59	Lievens *et al*. (2009)
*SIX4*	SIX4	TCAGGCTTCACTTAGCATAC/GCCGACCGAAAAACCCTAA	967	59	Lievens *et al*. (2009)
*SIX5*	SIX5	ACACGCTCTACTACTCTTCA/GAAAACCTCAACGCGGCAAA	667	59	Lievens *et al*. (2009)
*SIX6*	SIX6	CTCTCCTGAACCATCAACTT/CAAGACCAGGTGTAGGCATT	793	59	Lievens *et al*. (2009)
*SIX7*	SIX7	CATCTTTTCGCCGACTTGGT/CTTAGCACCCTTGAGTAACT	862	59	Lievens *et al*. (2009)
*SIX8*	SIX8	TCGCCTGCATAACAGGTGCCG/TTGTGTAGAAACTGGACAGTCGATGC	250	59	Meldrum *et al*. (2012)
*SIX9*	FOL SIX9	GGGTGGACCATATCACGATGTTCG/GAATACCTGAGTGGAGTTGTGTCTTG	458	69	This study
*SIX9*	FOC SIX9	GGCCCAGCCCTAGTCTAACTCC/AACTTAACATGCTGGCCGTCAATCG	347	67	This study
*SIX10*	SIX10	GTTAGCAACTGCGAGACACTAGAA/AGCAACTTCCTTCCTCTTACTAGC	636	65	This study
*SIX11*	SIX11	ATTCCGGCTTCGGGTCTCGTTTAC/GAGAGCCTTTTTGGTTGATTGTAT	559	61	This study
*SIX12*	SIX12	CTAACGAAGTGAAAAGAAGTCCTC/GCCTCGCTGGCAAGTATTTGTT	449	61	This study
*SIX13*	SIX13	CCTTCATCATCGACAGTACAACG/ATCAAACCCGTAACTCAGCTCC	1027	61	This study
*SIX14*	FOL SIX14	ATAAAGTGCGACTGGACTTCTGCC/ACCCCCATCCACATTCCTAAGCGA	422	67	This study
*SIX14*	FOL SIX14 nest	GATCCCAATGGGGGCTGTGT/GCTGGTGGCTAGAATCTCTTTGGA	232	59	This study
*SIX14*	FOC SIX14	ACAACACCGCGACGCTAAAAAT/GCACACTCAGTGCGACAAGTTC	438	61	This study
*C5*	C5	AGAGTGTGAAGTGAGGACGAGGGA/CTACGTTCGCCTCACTCATTGCCT	1064	63	This study
*CRX1*	CRX1	CACCATCTGTCTACATAAGGCCGCCC/AAAGTTCAAGGACCGGACCGCCG	1654	69	This study
*CRX2*	CRX2	TTAGTCGCACATCTACCATCACTG/GGAGTCGATCTAACTTCAGG	856	58	This study
*CRX2*	CRX2 FP	CCAGTGCATTGGTTTGAGACGTT/ATGCGCTCGCTTTCTATGTATCTG	902	63	This study
**Primers used for real‐time reverse transcription‐polymerase chain reaction (RT‐PCR)**					
*SIX3*	QSIX3	GGCCGTCTTCTACTTCATTTAC/GGGAGAATGTTCTAGCATAACC	69	63	This study
*SIX5*	QSIX5	TGGGCTCGAAAAGTCCAGCAT/TGTTTCGCCGTCAATGTCGCC	114	63	This study
*SIX7*	QSIX7	TCGATCTCTTTCCAAGACAAGGGCA/GTGGACGCGGCGTTGGTGAAC	130	63	This study
*SIX9*	QSIX9	GCCGACCCAGACCTACGCTTT/GCTGGTTTTGGAAGCCCAGTTGT	129	63	This study
*SIX10*	QSIX10	CCCGGAAAGCCTGCATCGACTA/AGAACAAACGTCGGTGGGACCA	53	63	This study
*SIX12*	QSIX12	TGCTGCTCCAAGTACAAACTACCTT/GCTGATACCTTTGGGTCCAACGC	71	63	This study
*SIX14*	QSIX14	ATGTCGTATGCCGGACGGGAA/TTATCTCGTAGACGCCTTCCT	109	60	This study
*C5*	QC5	GCCTATGGCAGGACTTGTTGAC/CCACAGCTTCTTGGACTATCTCC	126	63	This study
*CRX1*	QCRX1	AACTCAGGTACCACATCGGGA/CAGGTCGTCCTAGCGTCAGT	89	60	This study
*CRX2*	QCRX2	CAATCAGAAACCACGACGGAA/GGAGTCGATCTAACTTCAGG	89	60	This study
*EF‐1α*	QTEF	GGTCAGGTCGGTGCTGGTTACG/TGGATCTCGGCGAACTTGCAGG	77	63	This study
*TUB2*	QTUB	TTCTGCTGTCATGTCCGGTGT/TCAGAGGAGCAAAGCCAACCA	134	63	This study

*EF‐1α, *translation elongation factor 1α*; *RPB2*, *RNA polymerase II second largest subunit* ; *SIX*, *Secreted In Xylem*; *TUB2*, *β‐tubulin*.

### Molecular characterization of *Fusarium* isolates: putative effectors

Molecular characterization through the detection of the presence/absence and sequencing of putative effector genes was carried out for all *Fusarium* isolates in Table 1. Isolates were assessed for the presence/absence of *SIX1–SIX14* using published primers where available (for *SIX1–SIX8*) (Lievens *et al*., 2009; Meldrum *et al*., 2012), whereas new primers were designed for *SIX9–14* based on the published genome sequences of FOL (isolates MN25/4287, Table 4). Nested primers (FOL SIX14 nest) were used for *SIX14* because of the very short sequence reads and poor quality sequence data, and PCR was carried out as described below, using 1 µL of purified PCR product. FOL isolate MN25 was used as a positive control for all *SIX* genes, with the exception of *SIX4*, where isolate FOL1 was used, as MN25 does not contain *SIX4* (Broad Institute/MIT, 2007). All primer sequences were checked against target *SIX* sequences using a preliminary assembly of the FOC genome (Vágány, 2012) and, following this, new primers were designed for *SIX9* (FOC SIX9) and *SIX14* (FOC SIX14), because of the large sequence differences between FOC and FOL (Table 4). All PCRs for the *SIX* genes were set up as described for the housekeeping genes with standard thermocycling conditions as follows: one cycle of 2 min at 94 °C; 30 cycles of 45 s at 94 °C, 30 s annealing (see Table 4 for temperatures) and 1 min at 72 °C, followed by one cycle of 5 min at 72 °C. A *de novo* assembly of 70‐bp paired‐end reads (Illumina, San Diego, CA, USA, GAIIx sequencing) was carried out using Velvet to obtain a preliminary FOC genome for isolate FUS2 (Vágány, 2012; Zerbino and Birney, 2008), and resulted in 1511 contigs with an N50 of 184 kb (raw 70‐bp paired‐end Illumina reads submitted to the NCBI Sequence Read Archive under BioProject PRJNA287483). This assembly was investigated for putative new effectors, including *SIX* genes. Proteins predicted by Augustus (Keller *et al*., 2011; Stanke and Morgenstern, 2005), which were located on the same contig as any of the *SIX* genes and had at least two of the three main characteristics of effector proteins [short length, presence of a signal peptide as predicted by SignalP version 4 (Petersen *et al*., 2011), no homology to any other known protein] were considered. This led to the discovery of several putative effectors, including gene *C5*. Then, any predicted protein in the rest of the assembly that met these criteria was considered. This list was refined by screening for the presence of published motifs related to pathogenicity, including RxLR‐dEER (Rehmany *et al*., 2005), which led to the discovery of two very closely related genes, *CRX1* and *CRX2*. Primers were then designed for *CRX1*, *CRX2* and *C5* (Table 4), and PCR was carried out using the thermocycling conditions described for the *SIX* genes. Additional primers were also designed (CRX2 FP) to amplify *CRX2* from *F. proliferatum* (forward primer based on *F. oxysporum* because of a lack of sequence for *F. proliferatum*; reverse primer based on *F. proliferatum* using sequence from initial PCR with CRX2 primers) in order to assess the presence/absence of the RxLR domain. All PCR amplicons were purified as described previously, and sequencing was carried out using forward primers or both primers for longer amplicons. Where both primers were used, contigs were constructed as described previously. For *F. proliferatum* (isolates A8, A40 and SP1_2), contigs were constructed from sequences of both PCRs. Sequences were aligned and maximum likelihood trees were constructed for *SIX7*, *SIX9*, *SIX10* and *SIX12*, as described previously. The models used were Kimura‐2‐parameter, gamma distributed for *SIX7* and with invariant sites for *SIX9* (Kimura, 1980), Jukes–Cantor for *SIX10* and Jukes‐Cantor, gamma distributed for *SIX12* (Jukes and Cantor, 1969). All available homologous sequences (NCBI) were included and sequences were extracted from all available *Fusarium* genome data following blast searches (Broad Institute/MIT, 2007). Trees were not constructed for any of the other *SIX* genes because of the limited number of sequences.

### Expression of putative effectors

For the examination of expression *in planta*, onion seedlings were infected with isolate FUS2 as described in Methods S1 (see Supporting Information). RNA was extracted from the pooled root systems of five seedlings using Trizol® reagent (Life Technologies, Paisley, UK), any DNA was removed using DNase I (Sigma‐Aldrich) and first‐strand cDNA was synthesized using Superscript II reverse transcriptase (Life Technologies) following the manufacturer's guidelines. Real‐time RT‐PCR was performed in a Roche Lightcycler 480 using the Lightcycler 480 SYBR Green I Master mix (Roche, Burgess Hill, Sussex, UK), following the manufacturer's instructions. Primers were used at a final concentration of 0.4 µm with the annealing temperatures in Table 4. The cycling conditions were as follows: one cycle of 95 °C for 5 min, followed by 45 cycles of 95 °C for 10 s, 60/63 °C for 10 s and 72 °C for 10 s. Melt curve analyses were used to confirm a single PCR product. All samples were run in triplicate, standard curves were plotted for each gene and data were expressed as the quantity of the target gene relative to the geometric mean of *EF‐1α* and *TUB2*.

## Supporting information

Additional Supporting information may be found in the online version of this article at the publisher's website:

**Fig. S1** Partial nucleotide alignment of the *SIX5* gene from *Fusarium oxysporum* f. spp. *cepae* and *lycopersici* (isolate MN25, Broad Institute *Fusarium* database). Shaded bases differ from the predominant sequence type.
**Methods S1** Protocol for analysing expression of putative effector genes *in planta*.
