# Supplementary material for: Identification of pathogenicity‐related genes in Fusarium oxysporum f. sp. cepae
Source: Mol Plant Pathol. 2016 Feb 23;17(7):1032–47. doi: 10.1111/mpp.12346 (PMC4982077; doi:10.1111/mpp.12346)
Supplement: Supplementary file 1 — Fig. S1 Partial nucleotide alignment of the SIX5 gene from Fusarium oxysporum f. spp. cepae and lycopersici (isolate MN25, Broad Institute Fusarium database). Shaded bases differ from the predominant sequence type. Methods S1 Protocol for analysing expression of putative effector genes in planta. [file MPP-17-1032-s001.docx]

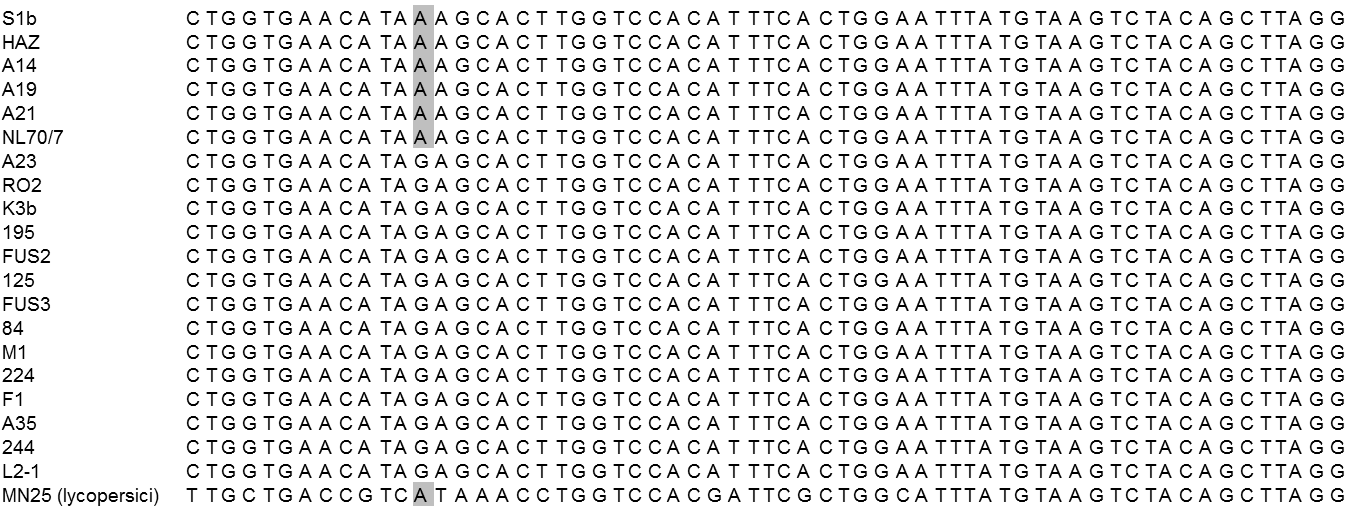


**Figure S1**. Partial nucleotide alignment of the *SIX5* gene from *F. oxysporum* f. spp. *cepae* and *lycopersici* (isolate MN25, Broad Institute *Fusarium* database). Shaded bases (position 316) differ from the predominant sequence type.

Supplementary method M1

Square petri dishes (10cm) were filled with autoclaved ATS medium (1M KNO_3_, 1M KPO_4_, 1M MgSO_4_, 1M Ca(NO_3_)_2_, 20mM Fe-EDTA, 70mM H_3_BO_3_, 14mM MnCl_2_, 0.5mM CuSO_4_, 1mM ZnSO_4_, 0.2mM Na_2_MoO_4_, 10mM NaCl, 0.01mM CoCl_2_, 0.45% Gelrite), and the top 5cm removed with a sterile scalpel. Onion seeds (cv. HZS) were sterilised in FICHLOR solution (3.1g sodium dichloroisocyanurate dihydrate in 50ml water, 1 drop Nonidet) by shaking for 6 min and after rinsing twice with SDW, 12 seeds were placed evenly across the cut surface and the plates sealed and incubated in a 15°C growth cabinet in the dark for 7 days for germination. The temperature was then increased to 25°C and the plates incubated for a further 7 days in light / dark (16 hour daylength). A spore suspension of *F. oxysporum* isolate FUS2 was prepared as described for the seedling tests, but with the addition of 200µl of tween per litre of SDW. Onion seedlings were then inoculated by evenly pipetting 1.8 ml of the spore suspension (1 x 10^6^ cfu ml^-1^) directly onto the roots. Plates were re-sealed and returned to 25°C. Root samples were taken at 8 time-points (0 (pre-inoculation), 8, 16, 24, 36, 48, 72 and 96 hours post inoculation). The whole root systems of 5 seedlings were removed, rinsed thoroughly in SDW, pooled and flash frozen in liquid N.
